# Local Genealogies in a Linear Mixed Model for Genome-Wide Association Mapping in Complex Pedigreed Populations

**DOI:** 10.1371/journal.pone.0027061

**Published:** 2011-11-02

**Authors:** Goutam Sahana, Thomas Mailund, Mogens Sandø Lund, Bernt Guldbrandtsen

**Affiliations:** 1 Department of Molecular Biology and Genetics, Faculty of Science and Technology, Aarhus University, Tjele, Denmark; 2 Bioinformatics Research Centre (BiRC), Aarhus University, Aarhus C, Denmark; Vanderbilt University Medical Center, United States of America

## Abstract

**Introduction:**

The state-of-the-art for dealing with multiple levels of relationship among the samples in genome-wide association studies (GWAS) is *unified mixed model analysis (MMA)*. This approach is very flexible, can be applied to both family-based and population-based samples, and can be extended to incorporate other effects in a straightforward and rigorous fashion. Here, we present a complementary approach, called ‘GENMIX (genealogy based mixed model)’ which combines advantages from two powerful GWAS methods: genealogy-based haplotype grouping and MMA.

**Subjects and Methods:**

We validated GENMIX using genotyping data of Danish Jersey cattle and simulated phenotype and compared to the MMA. We simulated scenarios for three levels of heritability (0.21, 0.34, and 0.64), seven levels of MAF (0.05, 0.10, 0.15, 0.20, 0.25, 0.35, and 0.45) and five levels of QTL effect (0.1, 0.2, 0.5, 0.7 and 1.0 in phenotypic standard deviation unit). Each of these 105 possible combinations (3 *h^2^* x 7 MAF x 5 effects) of scenarios was replicated 25 times.

**Results:**

GENMIX provides a better ranking of markers close to the causative locus' location. GENMIX outperformed MMA when the QTL effect was small and the MAF at the QTL was low. In scenarios where MAF was high or the QTL affecting the trait had a large effect both GENMIX and MMA performed similarly.

**Conclusion:**

In discovery studies, where high-ranking markers are identified and later examined in validation studies, we therefore expect GENMIX to enrich candidates brought to follow-up studies with true positives over false positives more than the MMA would.

## Introduction

Although most genome-wide association studies are based on single-marker tests, haplotype based tests are expected to hold more power, if properly applied [1]–[2]. Genealogy based haplotype tests are potentially the most powerful. Any potential phenotype-affecting mutation must have occurred on an ancestral lineage of a local genealogy, i.e. it must lie on an edge of a local gene-tree. If the local genealogies are known, they provide an optimal set of hypotheses to test, much smaller than if all haplotypes in a region were tested.

However, the true local genealogy is never known but must be inferred. There is generally a trade-off in inference methods between the accuracy and computational efficiency. The *Blossoc* method [3]–[5] mainly aims for computational efficiency. It readily analyses GWAS datasets in hours on a desktop computer, yet it still infers local genealogies sufficiently well to out-compete single marker tests in localization and ranking accuracy. An underlying assumption in *Blossoc*, however, is that the samples are unrelated, which is not always the case in human genetics and generally never for livestock populations.

The state-of-the-art for dealing with multiple levels of relationship among the samples is Yu *et al*. [6]'s *unified mixed model*. This approach is very flexible, can be applied to both family-based and population-based samples, and can be extended to incorporate other effects in a straightforward and rigorous fashion.

In this paper we present a new method, **GENMIX** (**gen**ealogy based **mix**ed model), which combines local genealogies with the unified mixed model. We compare it with the current state-of-the-art, unified mixed model [6], on simulated cattle data and show that GENMIX provides a better ranking of markers: in 90% of simulations, the highest ranked marker in GENMIX falls within 1Mbp of the true marker, compared to only 76% for the unified mixed model. In discovery studies, where high-ranking markers are identified and later examined in validation studies, we therefore expect GENMIX to enrich candidates brought to follow-up studies with true positives over false positives more than the unified mixed model would.

## Methods

The DNA was extracted from semen samples from Danish Jersey bulls for genotyping in a different project which has been acknowledged, so no ethical approval was required for this study.

### Pedigree and Marker Genotypes

We used the Danish Jersey population to simulate data. The marker genotypes of *Bos taurus* chromosome 6 (BTA6) of 1,407 individuals sampled from the pedigree of Danish Jersey dairy cattle were used for analyses. The pedigree was traced back as far as records were available (1937) and contained 8,063 individuals. Genotyping was done with the Illumina Bovine SNP50 BeadChip (Illumina Inc.) at the Aarhus University, Research Center Foulum, Department of Genetics and Biotechnology and at GenoSkan, AgroBusiness Park, Foulum, Denmark. Markers were assembled according to bovine genome assembly 4.0, Btau_4.0 [7]. Missing markers and linkage phase were inferred using the software fastPHASEnd [8]. SNP loci with a minor allele frequency of less than 5% were omitted. After data editing for genotyping quality, 1,695 SNPs were used for final analysis. The total bracketed length of the chromosome was 122 Mbp. The average distance between SNPs was 71.98 kbp.

### Simulated QTL and Phenotypes

We choose 7 SNPs randomly out of total 1,695 SNPs on BTA6 with minor allele frequencies 0.05, 0.10, 0.15, 0.20, 0.25, 0.35, and 0.45 as QTL. These 7 SNPs were spread across the chromosome and therefore, they represent a broad spectrum of regional LD. There were 5 levels of QTL effect 0.1, 0.2, 0.5, 0.7, 1.0 in phenotypic standard deviation unit. The phenotypes were obtained as the sum of a simulated QTL effect, a residual polygenic effect and a random residual. The residual polygenic effects were generated in two steps. First, polygenic values for the individuals with unknown parent were sampled from a normal distribution with mean 0 and variance of 1. The residual polygenic effects for the subsequent generation were derived by summing half of the values of the sire and dam residual polygenic values and a Mendelian sampling term. The residual variance was sampled to achieve three levels of heritability of 0.21, 0.34, and 0.64. These three levels of heritability were chosen to match the data from Yu et al. [6].

For each dataset one SNP, out of the seven SNPs selected based on MAF, was considered as a QTL. The SNP assigned as QTL was removed from the marker sets analyzed so the total number of markers used for each analysis was 1,694. The additive genetic variance due to the QTL was calculated as 2p (1 - p) α^2^; where α is allele substitution effect of the QTL and p is the minor allele frequency at the locus. In each simulation setting, α was adjusted based on the allele frequencies of the SNP in the pedigree to obtain each of the QTL explained a predefined proportion of the phenotypic variance. The phenotype of an individual was the total sum of the QTL effect, the residual polygenic effect and the random residual. The total number of analyses was 2,625 (3 levels of heritability x 7 MAFs x 5 effect sizes x 25 replications).

### Grouping Haplotypes Based on Location on an Edge of the Genealogy Tree

**Figure 1:**
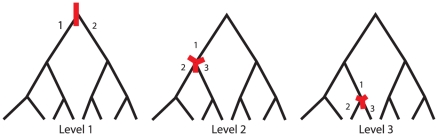
Illustration of generation of factor levels by bi- or trisection of the genealogies. The red mark illustrates the node where the genealogy is cut. Cutting at level 1 generates two haplotype clusters (1, 2), cutting at levels 2 and 3 generates three haplotype clusters (1, 2, 3). Cutting at levels 1, 2 or 3 generates 1, 2 and 4 clustering each, respectively.

The genome is first segmented into (overlapping) regions that can be explained by a single rooted binary tree topology, using the four-gamete test. For each such region, a tree explaining the genealogy of the region can then be constructed using the perfect phylogeny method very efficiently. Local genealogies were inferred by Blossoc [5], and translated in to explanatory factors for the linear model by bi- or trisecting the tree (Figure 1). The first level of tests was done by bisecting the tree at the root. All haplotypes descending from the same branch from the root node were grouped into one factor level. Subsequently, factors were generated by trisecting the tree at the second level and third level nodes. Each of these trisections resulted in three factor levels, one corresponding to haplotypes linking to the trisected node through the branch leading to the node's parent node, and two corresponding to haplotypes descending from the node through its branches to its offspring nodes. This generates a total of seven explanatory factors for each tree genealogy generated through Blossoc. We did not consider further down the tree (below third level) as the number of haplotypes in a lineage will decrease and might lead to numerical instability for analysis.

### Genealogy Based Mixed Model (GENMIX)

We split the tree at the top (one set of two clusters), the second level (two sets of three clusters) and at the third level (four sets of three clusters) as presented in Figure 1. Successively each clustering of haplotypes was included as a fixed effect in the model for analysis:
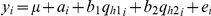
where *y_i_* is the phenotype of individual *i*, *μ* is the population mean, *a_i_* is the additive polygenic effect with *E(a_i_) = 0* and *Var(a) =* ***A*** *σ_a_^2^*, ***A*** is the numerator relationship matrix calculated based on pedigree records; *σ_a_^2^* is the additive polygenic variance; 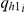 and 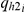 are the counts of the number occurrences of one or two of the haplotypes in individual *i*. For a bisection 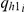 is the count of *h1_i_* (0, 1 or 2), *b_2_* being constrained to 0. The count of the other haplotype is 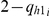. For a trisection (level 2 and 3; Figure 1) 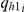 and 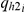 are the counts of haplotypes *h1_i_* and *h2_i_* (0, 1 or 2 subject to the constraint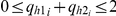), the count of the third haplotype in individual *i* being 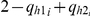. *b_1_* and *b_2_* are the substitution effects of the haplotypes. Finally, *e_i_* is a random residual. Variance component analyses were carried out using the software DMU (http://www.dmu.agrsci.dk/).

The significance of the SNP association was tested by testing whether the relevant regression coefficients are zero. This was tested using a Wald test. For testing the significance of a factor a vector of the two free factor levels, 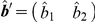 is obtained from the DMU output. REML estimates 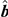 will asymptotically be distributed as



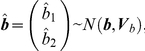
where ***b*** is the true value the regression coefficients and ***V*** *_b_* is the estimation variance-covariance matrix, also obtained from the DMU output. Under the null hypothesis 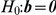 we have asymptotically that



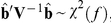
where *f* is the number of degrees of freedom. *f* = 1 for a bisection (*b_2_* being constrained to 0), *f = *2 for a trisection. The alternative hypothesis 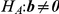 was tested against this. If the null hypothesis was rejected we have evidence for a non-zero effect when clustering according to this particular partitioning of the genealogy.

### Significance Tests

The significance threshold was determined using a Bonferroni correction for multiple testing. Testing was conducted at a nominal level of 5%. Test thresholds for individual tests were determined by correcting for 7 tests (1 bisection and 6 trisections) at each SNP. The smallest p value amongst the 7 tests for a SNP was considered QTL-SNP association. Correction was done for 1,694 SNPs. Thus the number of tests corrected for in the Bonferroni correction was 11,858. The significance threshold for the individual tests was there for 4.2×10^−6^.

### Unified Mixed Model Analyses

Following Yu et al. [6] a polygenic genetic effect was fitted as a random effect and single SNPs were successively included as fixed effect in the model. Significance of the haplotype substitution effect (*α)* each marker's was tested using a Wald-test against a null hypothesis H_0_: *α* = 0. The significant threshold was fixed at 5% level after Bonferroni correction for multiple testing for 1,694 simultaneous tests, resulting in a threshold on the individual test being 3.0×10^−5^.

## Results

### Ranking

**Figure 2:**
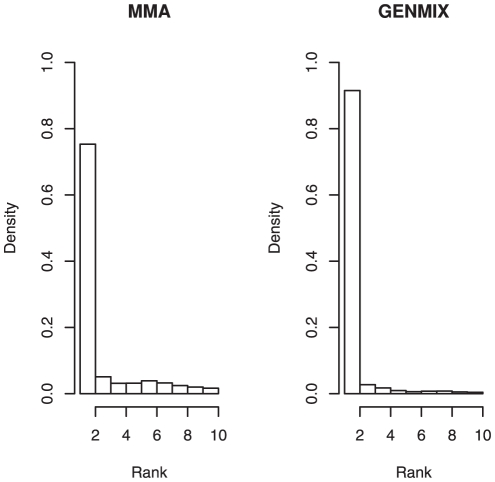
Distribution of the highest-ranking marker within a 1 Mbp radius of a quantitative trait nucleotide with MAF = 0.10 for 375 replicates (3 h^2^ x 5 QTL effects x 25 replicates).

**Figure 3:**
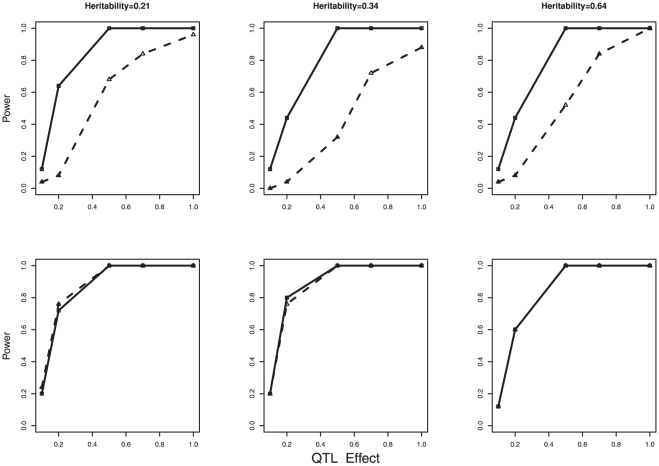
The power to detect QTL with different effect size for three different levels of heritability using GENMIX (□) and MMA (△). The QTL effects in phenotypic standard deviation unit are in the X-axis and the power to detect QTL is in the Y-axis. The first row is for MAF of 0.10 and the second row is for MAF of 0.45.

We tested the ability of GENMIX to rank the true positive markers against MMA. We define the rank of a marker as its position in a list of all the marker values sorted by ascending *p* values, the highest rank being assigned to the most significant markers. In case of two markers getting exactly the same rank, we randomly decide which of them gets the highest rank. Figure 2 shows the distribution of the highest rank marker within 1 Mbp of the QTL polymorphism for the simulated replications. There were 27 markers in the interval surrounding the QTL. The results in Figure 2 show that GENMIX is far more likely to have a highly ranked marker close to the QTL than MMA is. The GENMIX outperformed MMA with respect to ranking of close markers when MAF was low (e.g. 0.10 and 0.15). However, this difference in ranking performance was not observed for MAF 0.05, as the power to detect QTL for scenarios with MAF 0.05 was extremely low for both methods (Figure 3). Besenbachar et al. [3] had reported that genealogy based method (QBlossoc) was more likely to have a high scoring maker closer to the causative locus than single-marker analysis. In single marker based analysis, the chance of a marker with similar allele frequency with QTL (in strong linkage disequilibrium) resulting in strong association signal irrespective of its distance from the QTL is higher compare to haplotype-based analysis. Because, in haplotype based analysis a number of markers are considered jointly and it is highly unlikely that a haplotype located at a distance from the QTL will have similar allele frequency as the QTL and therefore, will not show strong association.

### Power

We considered a simulated QTL as having been detected if any haplotype within 2.5 Mbp was significant after Bonferroni correction. In general, GENMIX outperformed MMA when the QTL effect was small and the MAF at the QTL was low. In scenarios where MAF was high or the QTL affecting the trait had a large effect both GENMIX and MMA performed similarly. Powers of QTL detection for the two methods for three levels of heritability each for five levels of QTL effects are presented in the Figure 3 for MAFs 0.10 and 0.45. With MAF  =  0. 10 scenarios, GENMIX had higher power when the QTL effects were between 0.1 to 0.7 phenotypic SD. However, when MAF was 0.45, both methods performed similarly.

## Discussion

We have presented GENMIX, a genealogy-based method that achieves higher power and better ranking than the current state-of-the-art, the unified mixed model analysis introduced by Yu et al. [6]. GENMIX combines advantages from two powerful association-mapping methods: genealogy-based haplotype grouping and unified mixed model analysis. Genealogy-based methods perform better than single-marker analysis, as haplotype approaches can combine sets of common markers to identify a rare haplotype in strong LD with a rare causative variant [3], [5], [9]. The SNP density was relatively low (1 per 72 kbp) compared with most GWA studies in human and other species. Therefore, the haplotype grouping approach might not capture all the information contained in the local genealogy in this study. We expect that GENMIX may perform better than observed in the present study with the SNP higher density available now for many species. The mixed-model approach allows the incorporation of multiple levels of relatedness in the model instead of pre-correcting the data for pedigree. Even when exact relationships are unknown this combination of properties will stay advantageous as unknown relationships can be inferred based on the markers. Future large-scale association studies will analyze thousands of samples from multiple populations in an effort to detect common genetic variants of weak effect [10]. GENMIX provides a powerful approach to analyze such combined data. The GENMIX software is available on request to the authors.

### Running time

The computer time required to analyses a chromosome with 1000 marker using GENMIX was ∼2.5 h in a IBM HS22 blade servers equipped with one Intel Xeon X5570 2.93 GHz CPU and 48 GB RAM.
